# Gastric-type endocervical adenocarcinoma, superficial myofibroblastoma, sex cord-stromal tumors, and HSIL in Peutz−Jeghers syndrome: a rare case report, genetic characterization, and review of literature

**DOI:** 10.3389/fonc.2025.1472017

**Published:** 2025-02-13

**Authors:** Dongjin Sun, Yumei Li, Zhixing Cao

**Affiliations:** ^1^ Department of Pathology, Sir Run Run Shaw Hospital, Zhejiang University School of Medicine, Hangzhou, Zhejiang, China; ^2^ Department of Pathology, the Fifth Affiliated Hospital of Sun Yat-sen University, Zhuhai, China; ^3^ Department of Pathology, Zhuhai People’s Hospital, Zhuhai/Zhuhai Hospital Affiliated with Jinan University, Zhuhai, China

**Keywords:** somatic mutations, gene enrichment analysis, whole-genome sequencing, gastric-type endocervical adenocarcinoma, Peutz-Jeghers syndrome

## Abstract

Peutz-Jeghers syndrome (PJS) is characterized by an increased risk of gynecologic tumors. Gastric-type endocervical adenocarcinoma (GEA) is a rare non-human papillomavirus (HPV)-related tumor. We reported an uncommon case of a 39-year-old woman with PJS who developed GEA, superficial cervical vaginal myofibroblastoma, sex cord-stromal tumors with annular tubules of the ovaries, and cervical and vaginal high-grade squamous interepithelial neoplasia (HSIL). Before being verified GEA, the patient had been experiencing suspicious symptoms for over 9 years, with nabothian cysts and vaginitis being misdiagnosed. HSIL displayed widespread p16 immunostaining, and HPV DNA screening confirmed HPV-18 infection, although GEA was negative. Further, we verified *TP53* mutation and *HER2* amplification of GEA by fluorescence *in situ* hybridization (FISH). *TP53* was the most commonly mutated gene. The therapy with the anti-HER2 antibody trastuzumab was suggested based on HER2 amplification. We also analyzed the somatic mutations of GEA by whole genome sequencing (WES). There were 157 single nucleotide variations (SNVs) and 215 indels, with all of them being heterozygotes. Nonsynonymous and frameshift insertions were the most common kinds of mutations. The germine *STK11* gene mutation was found, which may play an important role in tumor development. According to gene function enrichment analyses, the genomic changes primarily implicated general transcription or expression pathways and cell cycle pathways. In addition, the JAK2/STAT3 pathway could be a major focus of targeted therapy for GEA patients with PJS. Our findings show that the patient with PJS can have a variety of unusual gynecologic tumors. Patients with PJS must have routine gynecological, ultrasonographic, and cytological examinations to detect precursor or early-stage lesions. The patient’s abnormal symptoms must be treated early with caution. A comprehensive genomic study reveals the potential causative genetic factors, therapeutic targets, and chemotherapy resistance of GEA. Further research will focus on the main driving genes, molecular mechanisms, and molecular target therapy in more patients.

## Introduction

Peutz-Jeghers syndrome (PJS) is a rare autosomal dominant disorder caused by germline mutations in the *STK11/LKB1* gene on chromosome 19p13.3 (1). The *STK11/LKB1* gene is involved in cell polarity, apoptosis, metabolism, and proliferation by activating the AMP-activated serine/threonine protein kinase (AMPK) pathway (1, 2). The reduction in *STK11/LKB1* expression in normal cells leads to the development of cancer (3). Patients with PJS are characterized by gastrointestinal hamartomatous polyps, mucocutaneous pigmentation, and have a high incidence of malignancies, especially in the lower gastrointestinal tract and female reproductive system (4). Gastric-type endocervical adenocarcinoma (GEA) of the cervix and sex cord-stromal tumors with annular tubules (SCTAT) of the ovary are common gynecological tumors (5).

GEA of the cervix is a rare tumor, which is a human papillomavirus (HPV)-independent adenocarcinoma that accounts for 10%-15% of all cervical adenocarcinomas (6). P16 immunoreactivity and HPV DNA detection are generally negative (7). Patients with PJS may be diagnosed with GEA and other multiple neoplastic lesions, including mucinous tumors of the fallopian tube or ovary, SCTAT of the ovary, and serous tubal intraepithelial lesions (5, 8).

To understand the pathogenesis and therapeutic targets of GEA, gene detection has been reported. By next-generation sequencing (NGS) performed on 21, 15, and 14 cases, *TP53* was found to be the most frequently mutated gene, followed by STK11 (9–11). The mutated genes are mainly involved in DNA damage repair, cell cycle, epithelial-mesenchymal transition (EMT), PI3K/AKT signaling pathways, and so on (9–11). Selenica et al. (12) performed massively parallel sequencing analysis on 68 GEA patients, identifying the most frequent pathways involved in the cell cycle (*TP53, CDKN2A*), PI3K-AKT (*PIK3CA, STK11*), and Notch (*FBXW7, NOTCH2, CREBBP, SPEN*). In the therapeutic target, ERBB3, ERBB2, and BRAF were detected. With the development of sequencing technologies, whole-exome sequencing (WES) was performed on 25 pairs of GEA tumors and matched normal samples, and the common *TP53*, *CDKN2A*, and *SKT11* were discovered, as well as the new somatic copy number alteration (SCNA) of *APOBEC3B* (13).

Here, we present a case of a PJS patient who also has GEA, superficial cervical vaginal myofibroblastoma (SCVM), and SCTAT of the bilateral ovary. SCVM was first reported in patients with PJS. More interestingly, we found high-grade squamous intraepithelial neoplasia (HSIL) in the cervix and vagina, and P16 was diffuse and strongly positive. Furthermore, we performed WES on paired GEA tumors and peripheral blood samples to explore the potential causative genetic factors and therapeutic targets of GEA with PJS, primarily through somatic mutation genes and functional enrichment analysis.

## Case presentation

### Clinical characteristics

**Figure 1:**
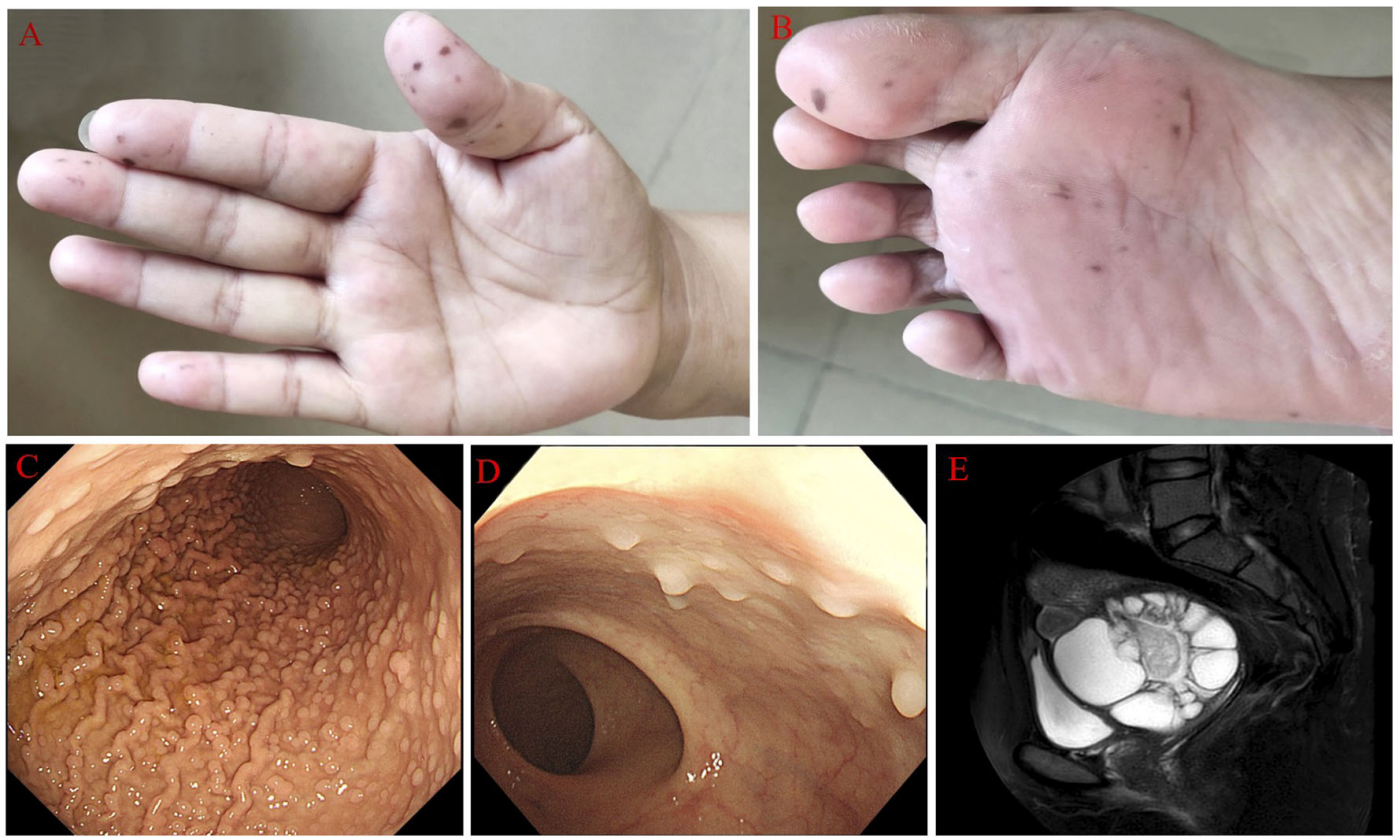
The main clinical characteristics of PJS patients. **(A, B)** Pig pigmentation on her digits and feet. **(C, D)** Multiple polyps in the stomach and intestinal tract. **(E)** Contrast-enhanced magnetic resonance imaging showed multiple cystic/solid lesions in the pelvis.

A 39-year-old female with mucocutaneous pigmentation on her digits, feet, and lips (**Figures 1A, B**) and multiple hamartomatous polyps in the gastrointestinal tract (**Figures 1C, D**) was diagnosed with PJS at the age of 21 years in 2004. She had undergone bowel resection due to intestinal obstruction caused by intestinal polyps in 2015. Her father had also been diagnosed with PJS. Her menstrual cycles prolonged to between 30 and 180 days approximately one decade ago. So far, she was not pregnant.

Sonographic findings revealed a significantly enlarged cervix (4.2×6.7×6.4 cm), and Nabothian cysts were considered during a physical examination 9 years ago. This situation was not emphasized. The patient complained of thin and mucoid vaginal discharge and vaginal odor, but this situation had been diagnosed with vaginitis and treated correspondingly many times 7 years ago. However, her symptoms were hardly relieved and recurred frequently. In this period, a plain CT scan showed larger cervical cysts than before in the lower abdomen, but further treatment was not performed. In 2021, a little hemorrhage after intercourse occurred for 2 to 3 days. Sonographic findings suggested a pelvic cystic mass, but a further measurement had not been made. The woman came to our hospital in 2022 with a vaginal hemorrhage (no clear reason). A gynecological examination revealed a thin, watery discharge and an enlarged cervix, as well as an 8×7×8 cm cystic mass posterior to the uterus. Contrast-enhanced magnetic resonance imaging (MRI) in the lower abdomen revealed multiple cystic/solid lesions with sizes of 8.6×9.5×9.1 cm in the pelvis (**Figure 1E**), which were closely related to the cervix. Sonography showed an 11.4×8.6×11cm area surrounding the cervix with uneven anechoic patches, suggesting hydrosalpinx. Both sides of the ovary were indistinct. The serum levels of CA125 and CA199 were within normal limits.

The biopsy of the cervical tumor and pelvic cyst via laparoscopic surgery was certified as HPV-unrelated GEA; however, the polyp at the junction of the uterine body and cervix was diagnosed as superficial cervicovaginal myofibroblastoma.

Subsequently, PET-CT indicated the residual tumor tissue and suspected lymph node metastasis in the pelvic cavity. The FIGO stage was classified as II B according to the clinical staging standard of the International Federation of Obstetrics and Gynecology (FIGO, 2018). The patient underwent a total hysterectomy, bilateral uterine adnexectomy, pelvic lymphadenectomy, and pelvic adhesionlysis in May 2022. The maximum invasion depth was around 2.9 cm, with cancer tissues visible on the uterine serosal surface, paracervical region, and no lymph node metastasis. The patient received neoadjuvant chemoradiotherapy, including cisplatin/paclitaxel combination chemotherapy.

In December 2023, the patient was readmitted to the hospital because of abdominal pain, distension, and nausea. PET-CT revealed disease progression, including several regional lymph node metastases as well as metastases in the abdominal cavity, pelvic cavity, and abdominal wall. She also developed severe small bowel fistulae. Finally, she died as a result of tumor recurrence and serious complications, despite receiving Candonilimab therapy. The disease-free survival time of this patient after receiving surgery and chemoradiation therapy was about 19 months.

### Pathological examination and FISH analysis

**Figure 2:**
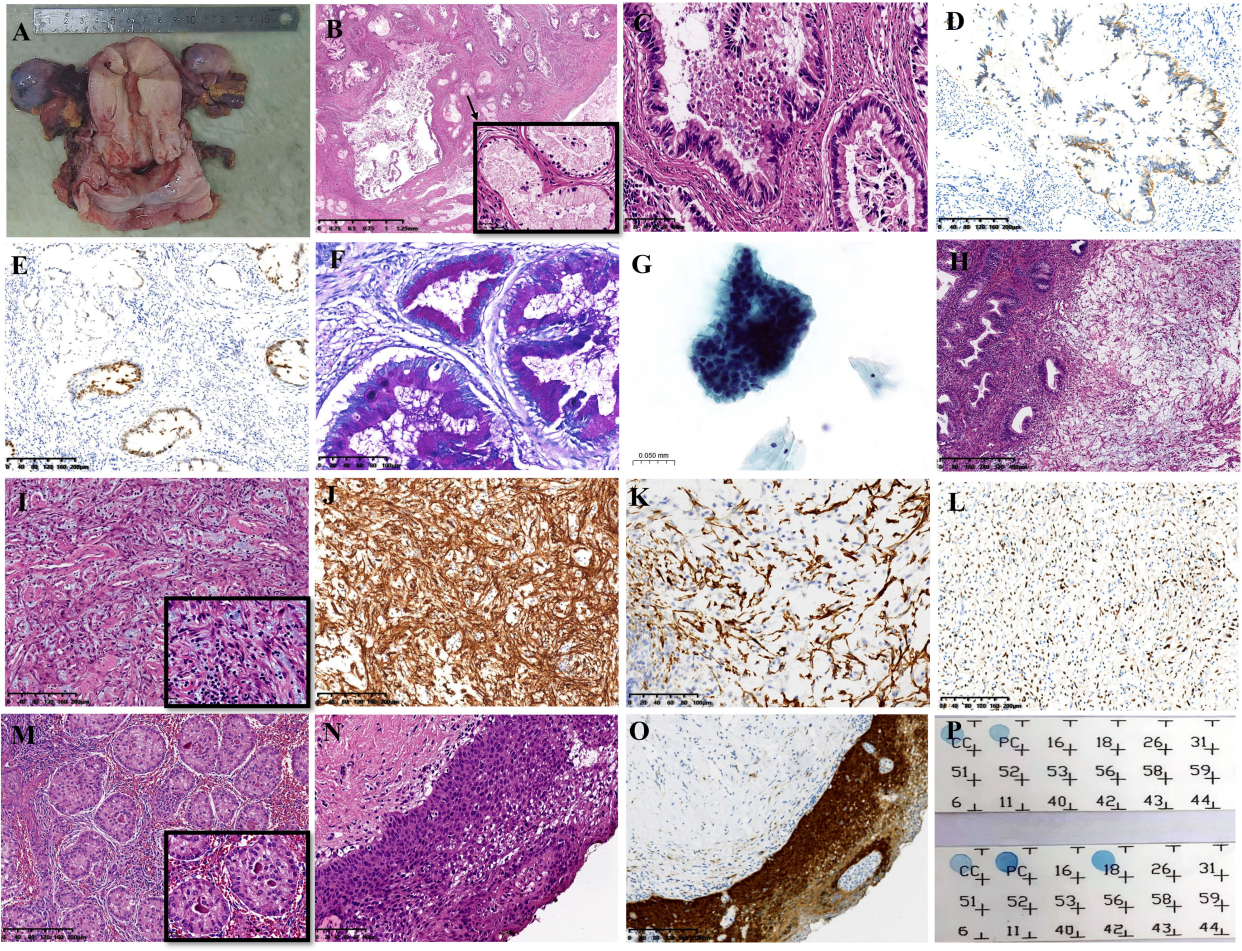
The distinct features of multiple tumors. **(A)** The gross feature showed a barrel-shaped cervix. **(B)** GEA was composed of variable shapes and sizes of glands (HE: ×16). **(C)** The tumor cells had prominent eosinophilic cytoplasm, hyperchromatic and enlarged nuclei, localized necrosis, and goblet cell development in certain regions (HE: ×200). **(D)** Positive staining for HER2 by immunohistochemistry (IHC: ×100). **(E)** P53 staining (IHC: ×100). **(F)** AB-PAS staining showed pink−red (×200). **(G)** Cytology test results (HE: ×400). **(H)** SCVM was under normal glands (HE: ×50). **(I)** Small to medium-sized vascular proliferation with hyaline degeneration (HE: ×100). **(J)** Focal positivity of Desmin staining (IHC: ×100). **(K)** Diffuse positivity of CD34 staining (IHC: ×200). **(L)** The positive index of PR was approximately 30% (IHC: ×100). **(M)** SCTAT in bilateral ovaries (HE: ×100). **(N)** HSIL of the vagina (IHC: ×200). **(O)** Diffuse positive immunoreaction of p16 in HSIL (IHC: ×100). **(P)** HPV DNA detection results. The upper panel displays the negative GEA results; the lower panel displays the HPV-18 infection of HSIL. GEA: A-G; SMFGT: H-L.

**Figure 3:**
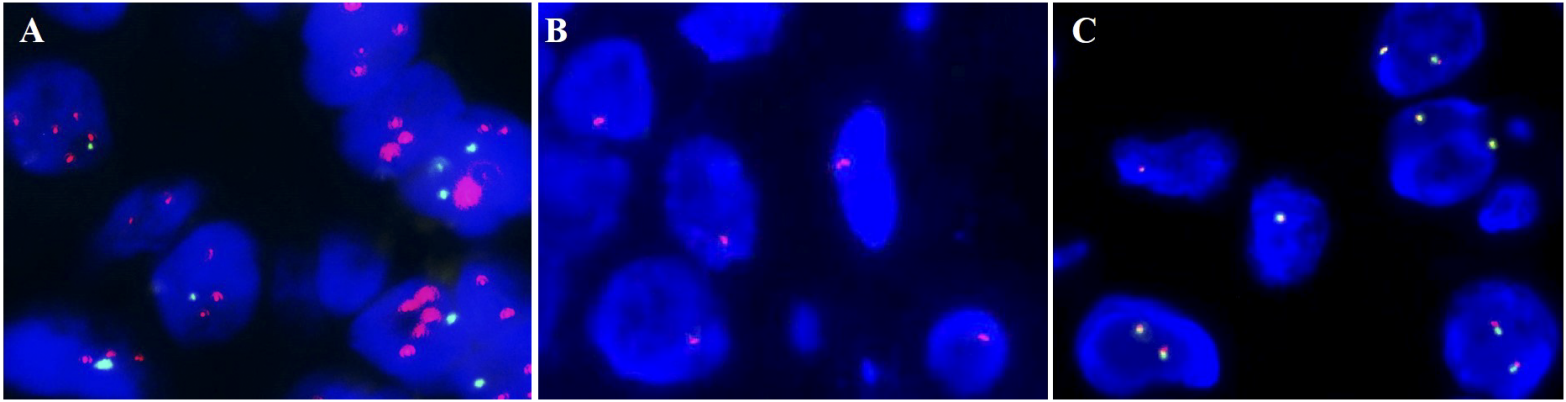
Fluorescence *in situ* hybridization (FISH) results. **(A)** *HER2* amplification. **(B)** The 1 red signal represents the heterozygous deletion; the 0 red signals suggest the homozygous deletion of *RB1*. **(C)** *HMGA2* rearrangement was negative.

A gross specimen of gastric cancer displayed the barrel-shaped morphology of the cervix (**Figure 2A**). Microscopically, multiple cysts and glands of varying sizes in fibrous tissues were lined by columnar cells with abundant, pale, or weak eosinophilic cytoplasm and clear cell boundaries, and no significant cytological atypia was observed in larger regions (**Figure 2B**). In certain areas, the tumor cells displayed hyperchromatic and enlarged nuclei, as well as necrosis, strong eosinophilic cytoplasm, goblet cell differentiation, and abortive mucin production (**Figure 2C**). The tumor cells showed positive expression of MUC-6, CAIX, MUC5AC, HNF-1β, MUC-1, CK7, and Pax-8. P16 immunoreactivity was focal and weak. The Ki67 proliferation index was approximately 15%. It was negative for ER, PR, PAX-2, CK20, CDX-2, and NKX3.1. Mismatch repair proteins, including PMS2, MSH2, MSH6, and MLH1, have expression indices ranging from 10% to 90%. HER2 staining was determined as 2+ in some areas when evaluated by IHC (**Figure 2D**). Furthermore, *HER2* amplification was identified by FISH, and the ratio of red to green signals was approximately 2.42. (**Figure 3A**). P53 was categorized as a mutation type because its positive index was almost 70% in the region with the most atypia (**Figure 2E**); otherwise, it was wild-type in the MDA regions by IHC. The tumor cells stained pinkish-red with AB-PAS, indicating neutral mucus (**Figure 2F**). The tumor’s HPV DNA test was negative (**Figure 2P**). A few glandular cells with plentiful cytoplasm and mild to moderate atypical hyperplasias were detected by the cervical liquid-based cytology test (**Figure 2G**), but due to the well-differentiated cell structure, it was easily overlooked.

There was a polypoid protrusion with stellate cells and long spindle cells at the uterocervical junction under the endometrium and cervical glands (**Figure 2H**). These cells had mild to moderate nuclear atypia and red cytoplasmic staining. Intercellular thin collagen fibrils, mucinous degeneration, lymphocyte and mast cell infiltration, and small- to medium-sized vascular proliferation with hyaline degeneration were among the interstitial histologic characteristics (**Figure 2I**). Desmin had localized positivity (**Figure 2J**). The diffuse, strong positive was exhibited by vimentin, SMA, and CD34 (**Figure 2K**). Ki67 had a positive rate of approximately 5%. ER and PR immunostaining was observed in approximately 30% of the cells (**Figure 2L**). Mast cells were identified by CD117. The expression of Rb was absent. Subsequently, we confirmed the monoallelic deletion of *13q14(RB1)* (**Figure 3B**), and FISH results for the *HMGA2* broken gene were negative (**Figure 3C**). Our analysis revealed that it was SCVM.

Under microscopy, SCTAT linked to PJS was discovered in the bilateral ovaries. The hyaline basement membrane-like substance was surrounded by tumor cells, which were organized in rosette-like patterns (**Figure 2M**). The tumor cells expressed calretinin, WT-1, CD99, and inhibin-α.

The accompanying lesions included endometrial adenomyomatous polyps and HSILs of the cervix and vagina (**Figure 2N**). P16 displayed diffuse positivity for HSIL (**Figure 2O**), and infection with high-risk HPV-18 was discovered by HPV DNA testing (**Figure 2P**).

### Whole-exome sequencing and data analysis of GEA

We took a sample of control peripheral blood and formalin-fixed paraffin-embedded (FFPE) GEA tissues from this patient and performed WES with sequencing depths of 70.74× and 138.12×, respectively.

On tumor tissues, we discovered the germline STK11 gene nonframeshift deletion on exotic 5 on Chr19. Additionally, the tumor displayed common *TP53* and *ERBB2* nonsynonymous SNVs. We verified *HER-2* amplification by FISH, and P53 immunohistochemistry revealed the mutation type in some areas. In addition, we discovered some hotspot SNVs, including *POLE*, *BRCA1*, *BRCA2*, *CDX-2*, *MUC-6*, *CAIX*, *EGFR*, *APC*, *PDGFRa*, *BCL-6*, *USP6*, *Fli-1*, *NOTCH1*, *NOTCH2*, *PAX-5*, *TTF-1*, *ALK*, and *ROS1*, as well as Indels, such as *MUC-6*, *MUC-4*, *MUC-2*, *MAML-2*, and AR (**Supplymentary Tables S1**, **S2**).

### Identification of somatic mutations

**Figure 4:**
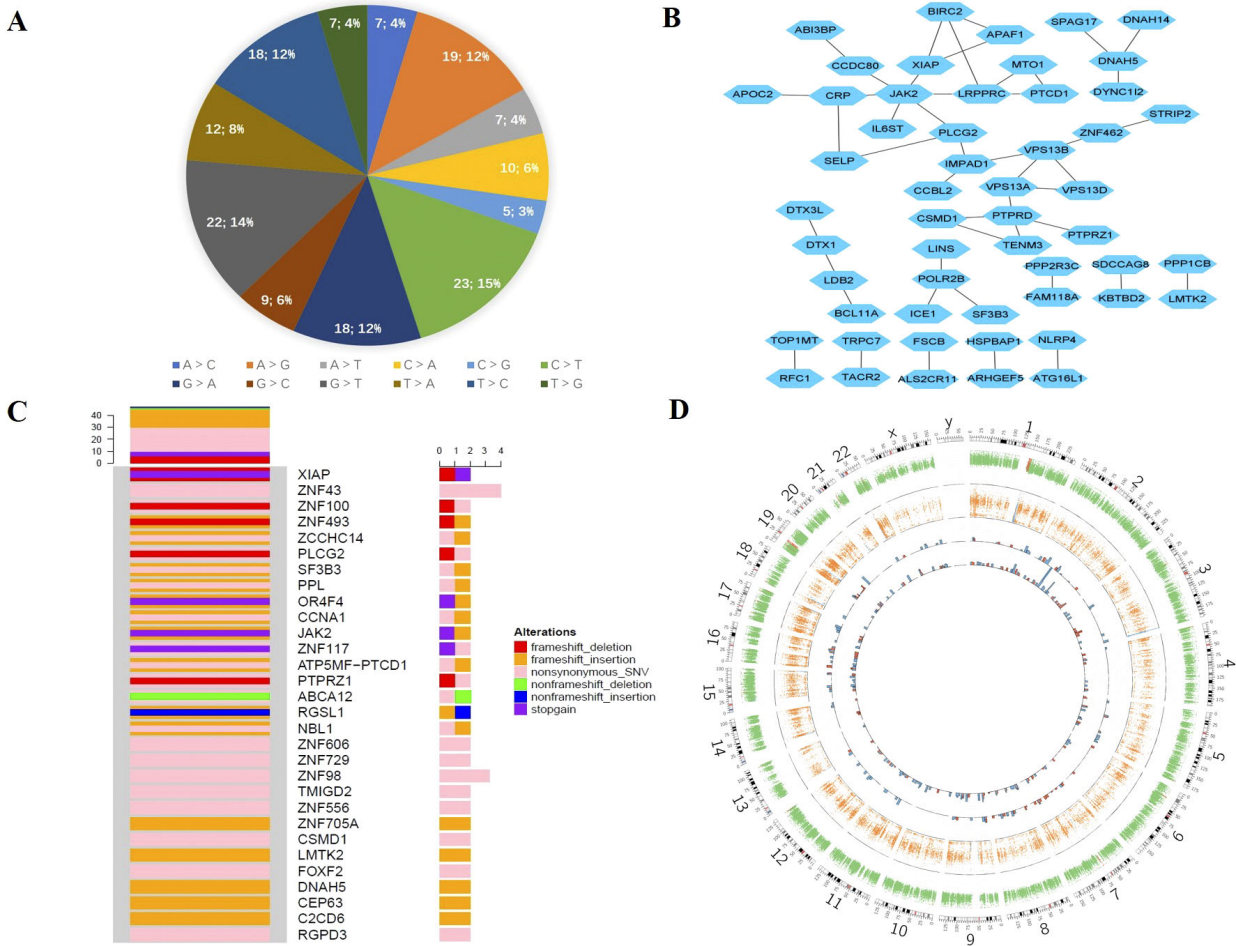
Somatic mutation of GEA. **(A)** The proportion of base substitutions in somatic mutations. **(B)** The network interaction of the main somatic mutation genes. **(C)** Oncoprint for the genomic mutation profile. **(D)** Circos plot of somatic mutations displaying the mutation frequencies, distributions, and CNV results. From the outside to the inside: the first circle represents the genome karyotype (24 chromosomes and the corresponding number); the second circle represents somatic CNV; the third circle represents LOH; the fourth circle represents somatic mutation SNV; and the last circle represents somatic mutation Indel. The red histogram column indicates mutations with functional effects, including stop gain, nonsynonymous, and frameshift effects; the blue histogram column indicates mutations without functional effects. The mutation frequencies are represented by the height of the column.

We sequenced and analyzed the patient’s tumor tissues and the control peripheral blood sample at the same time. Mutect2 and Varscan2 were utilized to identify somatic mutations and copy numbers. The number of passed SNVs and indels was 157 and 215, respectively, with all of them being heterozygotes (**Supplementary Table S3**). The tumor mutation burden (TMB) value was 2.98 mutations/Mb. We also examined the spectrum of base substitutions. C to T and G to T were the frequent transversions in somatic mutations, accounting for 15% and 14%, respectively. All percentages are displayed in **Figure 4A**.

The mutant-filter variants were obtained by screening the protein-coding areas for mutations and eliminating synonymous and unidentified mutations. It was chosen to include 149 mutations, including 59 nonsynonymous SNVs, 49 frameshift insertions, 17 frameshift deletions, 12 stop-gain mutations, 6 nonframeshift insertions, 5 nonframeshift deletions, and 1 stop-loss mutation. Nonsynonymous and frameshift insertion mutations, which made up approximately 40% and 31% of the total, were the most common forms. Furthermore, we obtained 116 genes from trustworthy sites and used Cytoscape to create the network interaction, which is shown in **Figure 4B**. The top core gene in the network interaction was JAK2.

We evaluated mutation frequency statistics on genes that passed somatic mutations in the tumor and then chose genes with a relatively high mutation frequency to draw the oncoprint. **Figure 4C** displays the mutation types and rates for the genes with mutation sites higher than or equal to 2. The majority of genes have two different types of mutations. Nonsynonymous SNV was the most prevalent mutation type, followed by frameshift insertion and nonsynonymous SNV. There were four SNV mutation sites in ZNF43.

We evaluated the somatic mutation using the Circos image, which exhibited the mutation’s frequency and distribution, as well as the somatic copy number variation (CNV) details (**Figure 4D**). The inner tracks display the SNV and Indel distribution in various chromosomes. The number of indels was higher than SNVs, and the majority of the mutations were found in chr1, chr2, chr7, and chr16, as indicated by the red histogram, which represents the functional alterations.

### Gene enrichment analysis

We performed a gene ontology enrichment study utilizing the passed somatic mutation genes via the gene ontology database from molecular function, cellular component, and biological process (**Supplementary Figure 1A**). The dynein complex was the most important cellular component, followed by the biological process of protein localization to the Golgi apparatus. Many associated genes were enriched by the biological control of biosynthetic processes, ion binding, cellular biosynthetic processes, RNA biosynthetic processes, and the molecular function of cation binding and metal ion binding. The degree of gene enrichment in phospholipid efflux was greatest when the rich factor was approximately 0.25.

Furthermore, we performed pathway enrichment using the KEGG pathway database, Reactome pathway database, BioCyc pathway database, and PANTHER (**Supplementary Figure 1B**). The generic transcription pathway was the most noticeable. There were approximately 40 genes in the pathway for gene expression. The enrichment degree of differential genes on apoptotic factor-mediated response and downregulation of the *ERBB4* signaling pathway was the highest. In the top 30 pathways, gene expression (transcription), cell cycle pathway, programmed cell death, signal transduction, and biosynthesis pathways played essential roles. Other affected pathways included developmental biology, the immune system, transport of non-small molecules, cellular processes, lipid metabolism, the circulatory system, and drug resistance. In addition, we also found the Notch signaling pathway (*DTX3L*, *DTX1*, *MAML1*), TNF signaling pathway (*BIRC2*), Ras pathway (*PDPK1*, *RAF1*, *PAK2*), p53 pathway (*APAF1*, *PDPK1*, *CDKN2A*, *PIK3C2B*), NF-kappa B signaling pathway (BIRC2, XIAP, PLCG2), JAK/STAT signaling pathway (*JAK2*, *RAF1*, *IL23R*, *IL6ST*), and PI3K-Akt signaling pathway (*JAK2*, *PDPK1*, *PPP2R3C*, *LAMA1*, *PPP2R2B*, *RAF1*, *PPP2R5A*). Some pathways were related to pancreatic cancer (*RAF1*, *CDKN2A*, *ARHGEF6*) and endometrial cancer (*PDPK1*, *RAF1*) (**Supplementary Table S4**).

In addition, we explored the disease enrichment in the patient’s somatic mutation genes using the GWAS Catalog, NHGRI GWAS Catalog, OMIM, and KEGG disease databases (**Supplementary Figure 1C**). The response to taxane treatment (docetaxel) was quite significant. Schizophrenia, schizoaffective disorder, bipolar disorder, and myeloproliferative neoplasms showed relatively higher degrees of gene enrichment.

In addition, we used MSIsensor to examine somatic microsatellite variation and determined that this tumor was the non-MSI-H type.

## Discussion

In this study, we thoroughly investigated a patient with PJS who also had GEA, bilateral ovary SCTAT, SCVM, and vaginal HSIL from clinical, histological, and molecular perspectives.

GEA often appeared in the middle and upper regions of the endocervical canal, with bigger masses producing a “barrel-shaped” cervix with no evident exterior lesions. The tumor cells of well-differentiated areas have no significant cytological atypia and are classified as minimum deviation adenocarcinoma (MDA), which is regarded as a highly differentiated type within the differentiation spectrum of GEA, but the term MDA is no longer recommended in WHO 2020 (14). These characteristics can also easily lead to missed diagnoses. A pattern of infiltrative growth, an irregular glandular structure, and neural mucus in pale or weak eosinophilic cytoplasm indicate GEA (5). The neural mucus, which is gastric mucus, is revealed by the pinkish-red staining of AB-PAS. ER, PR, P16, and Pax-2 are commonly negative (15). However, only a few instances display widespread p16 expression despite negative oncogenic HPV molecular testing, showing that diffuse p16 immunoreactivity is not necessarily an indication of a high-risk HPV-related tumor (16). The loss of Pax-2 expression can help to exclude lobular endocervical glandular hyperplasia (15, 17). MUC-6, MUC5AC, CAIX, and HNF-1 were immunoreactive, and we discovered germline mutations of *MUC-6* and *CAIX* in tumor tissue. When only a few well-differentiated glands are seen in cytology and endocervical curettage specimens, the GEA is easily ignored, and the useful clue is the pale and abundant cytoplasm.

We detected the germline alterations STK11 gene non-frameshift deletion in our GEA tumor by WES. Germline mutations of the STK11 gene are the leading cause of PJS and were detected in around 80% of patients (1). According to one study, endogenous *STK11/LKB1* knockdown accelerated cell cycle progression from G1 to the S phase, which was at least partially mediated by the decrease in the p53 and p16 pathways (3). We detected *STK11* gene nonframeshift deletion in our GEA tumor. This mechanism may explain the loss or decline of P16 expression in GEA and negative HPV DNA detection results. But we found cervical and vaginal HISL with diffuse P16 immunostaining and HPV-18 infection in our case. Several researchers have also observed this occurrence (18). The rarity of GEA coexisting with HPV infection emphasizes the importance of continuing research and close monitoring in the field of gynecological oncology.

Women with PJS have an increased risk for gynecologic tumors (2, 5). Our patient with PJS was diagnosed with GEA, SCVM, and SCTAT of the bilateral ovary. SCVM is a rare benign mesenchymal tumor. Magro et al. (19) validated the chromosome abnormalities linked to the loss of the 13q14 area in the lower female genital tract myofibroblastoma. We also found the RB1(13q14) deletion in SCVM. SCVM has been identified to be unrelated to HPV infection (20), and it has a tumor-hormone relationship due to the presence of estrogen and progesterone receptors. SCTAT is also an uncommon and distinct tumor that is present in approximately one-third of PJS patients (21). SCTAT with PJS is typically bilateral and visible under a microscope (21), with just a few instances indicating malignant tendencies (22, 23). However, SCTAT without PJS is frequently unilateral, massive, and malignant (21). The primary treatment for these tumors is surgical removal (20, 21).

Using immunohistochemistry and FISH, we first identified the *TP53* mutation and *ERBB2* amplification. Further, we also found germline alterations in the *TP53* and *ERBB2* genes by WES. *TP53* was the gene that was most often altered in GEA tumors. A study discovered that *HER2* overexpression and amplification were linked to poor progression-free survival, whereas mutant-type *p53* has no prognostic value (24). Some researchers have also observed *HER2* amplification, indicating that therapy with a monoclonal antibody against HER2 may be a potential therapeutic option for GEA expressing HER2 (24–26). Ehmann et al. (27) proposed excellent clinical outcomes with adjusted trastuzumab. Besides, one trial demonstrated that trastuzumab exhibited durable responses in heavily pretreated patients with HER2-expressing solid tumors, including cervical cancers (28).

Furthermore, we performed a somatic mutation analysis of GEA with PJS by comparing the patient’s GEA tumor tissues and the control peripheral blood sample. In this patient, we detected 372 mutations, all of which were heterogeneous at the mutational level. Nonsynonymous and frameshift insertions were the most frequent types of mutations. Liao et al. (13) performed WES on 25 Chinese patients with GEA and compared the prior genetic profiles. The most altered genes were found to be *TP53*, *CDKN2A*, *STK11*, *BRCA2*, *SMAD4*, *ERBB2*, *FRMPD4*, *TELO2*, *NOP2*, *ELF3*, *FGF1*, *DNAL1*, and *NRIP3*, and the enrichment of the *APOBEC* signature was significant. In our case, we discovered *CDKN2A* and *KMT2D* somatic mutations. *CDKN2A* is a common mutation that affects the cell cycle. The *KMT2D* mutation has been described in two GEA cases thus far, both of which were gastric adenocarcinomas with a poor prognosis and involved cell proliferation and differentiation (9).

In our case, *JAK2* was the top core gene in network interaction by Cytoscape, and it was widely dispersed in the cytoplasm of somatic cells. The activated *JAK2/STAT3* pathway is involved in the pathologies of cancer and inflammatory diseases, and it promotes carcinogenesis, tumor development, cancer cell survival, and the spread of solid tumors (29). In addition, it can be involved in mediating resistance to traditional cancer therapy, including cervical cancer (29). The STK11/LKB1 promoter contains a STAT binding/interferon gamma-activated sequence, and PRL-mediated increases in promoter activity require signaling through STAT3 and STAT5A, also involving JAK2 (2, 30). Some researchers claimed that IL-11-mediated activation of JAK/STAT3 was crucial in gastrointestinal carcinogenesis following *LKB1* mutations and that targeting this pathway offers therapeutic potential in PJS (31). However, the mechanism of the JAK2/STAT3 pathway in GEA remains unknown, which could be a key focus of targeted therapy for GEA patients with PJS.

In gene enrichments, generic transcription or expression pathways and cell cycle pathways played important roles in the pathogenesis of GEA. This result was consistent with Selenica’s findings (12). The response to taxane treatment (docetaxel) was the most significant in disease enrichment, which might indicate chemotherapy resistance. Park et al. [9] came to the view that EMT-related pathways may have a role in GEA aggression and chemoresistance. Some researchers (9–13) found the unique genomic changes of GAS, mainly involving cell cycle and PI3K/AKT signaling pathways. Currently, a phase II interventional trial is underway to investigate the safety, tolerability, and effectiveness of WX390, a PI3K-mTOR cell cycle inhibitor, in combination with toripalimab, a PD-L1 inhibitor, in patients with advanced GEAS and confirmed STK11 mutations (32, 33).

In our case, the GEA tumor was the non-MSI-H type. Garg et al. (11) showed MSH6, MLH1, MSH2, and PMS2 aberrations, but mismatch repair marker expression was intact in every instance. Only one Lynch syndrome patient had MSH6 staining deletion as of yet (16). Most likely, GEA did not arise via the MMR pathway.

GEA patients with PJS have poor outcomes, short progression-free survival (PFS), and overall survival (OS) (5, 34). Nine years ago, our patient had an ultrasound that showed an enlarged cervix, and seven years earlier, she complained of vaginal discharge. However, nabothian cysts and vaginitis were incorrectly identified as the cause of these symptoms. Our patient was diagnosed with IIB GEA after nine years. GEA can easily be misdiagnosed and missed due to the lack of typical clinical manifestation, hidden lesion location, negative high-risk HPV screening, and mild cytological atypia. Clinicians and pathologists must furthermore enhance a high level of awareness and vigilance for GEA. Clinicians should pay high attention to patients with long-term abnormal vaginal discharge. For patients with high suspicion of GEA, particularly PJS patients, the cervical multiple-point biopsy or conization of the cervix should be performed (35). PJS patients should have annual gynecological ultrasonography, gynecological exams, and cervical cytologic (including sexual life history) testing to detect precursor or early-stage lesions beginning at the age of 18-20, according to National Comprehensive Cancer Network (NCCN) guidelines. Given its rarity and little information on treatment results, the best therapeutic technique for GEA is unknown. Experts now advocate adhering to the most recent NCCN guidelines for cervical carcinoma (32). For early-stage high-risk disease, these guidelines recommend primary radical hysterectomy and pelvic lymphadenectomy ± para-aortic lymph node sampling and advise against fertility-sparing surgery. They also recommend removal of the appendix, greater omentum, bilateral adnexa, and any metastatic lesions in the pelvic and abdominal cavity. In addition, postoperative concurrent chemoradiation was administered to high-risk patients, defined as those with at least one major risk factor, including positive nodes, parametrial involvement, and positive surgical margin (Chinese Expert Consensus on Clinical Diagnosis and Treatment of gastric-type endocervical adenocarcinoma, 2023 edition). Our case followed these rules. Surgical resection or radiation combination was an effective form of treatment. However, chemotherapy may not be a suitable adjuvant therapy for GEA (9, 34).

We thoroughly investigated this complex case. However, there are certain limitations to this study. We were unable to evaluate the GEA driver genes with PJS since we only sequenced one case by WES and detected two equivalent somatic mutations compared to the previous genetic profiles. At present, our case is the first to report SCVM with PJS; more instances are needed to investigate the link between unusual SCVM and SCTAT with PJS.

In conclusion, it was extremely rare for GEA with PJS to occur alongside the rare SCVM, bilateral ovarian SCTAT, and cervical and vaginal HISL with infection with high-risk HPV 18. Patients with PJS require routine gynecological, ultrasound, and cytological tests to detect precursor or early-stage lesions. Gynecologists must handle PJS patients carefully to obtain early clinical detection and diagnosis. We also thoroughly investigated the GEA gene characteristics using WES. The *STK11/LKB1* mutation may play an important role in tumor development. *TP53* and *HER2* mutations were found. The patient is most likely to benefit from the use of the anti-HER2 antibody trastuzumab in the future. The somatic mutations mainly involved general transcription or expression pathways and cell cycle pathways, which revealed the potential causative genetic factors and chemotherapy resistance of GEA. In addition, the JAK2/STAT3 pathway could be a major focus of targeted therapy for GEA patients with PJS. However, more cases are needed to investigate the pathogenesis of GEA with PJS.

## Data Availability

The datasets presented in this study can be found in online repositories. The names of the repository/repositories and accession number(s) can be found in the article/**Supplementary Material**.

## Supplementary material

Supplementary Figure 1 Gene enrichment analysis in GEA. **(A)** Gene ontology enrichment analysis results. **(B)** Pathway enrichment analysis results. **(C)** Disease enrichment analysis results.
