# Supplementary figures and images for: Gastric-type endocervical adenocarcinoma, superficial myofibroblastoma, sex cord-stromal tumors, and HSIL in Peutz−Jeghers syndrome: a rare case report, genetic characterization, and review of literature
Source: Front Oncol. 2025 Feb 13;15:1472017. doi: 10.3389/fonc.2025.1472017 (PMC11865206; doi:10.3389/fonc.2025.1472017)

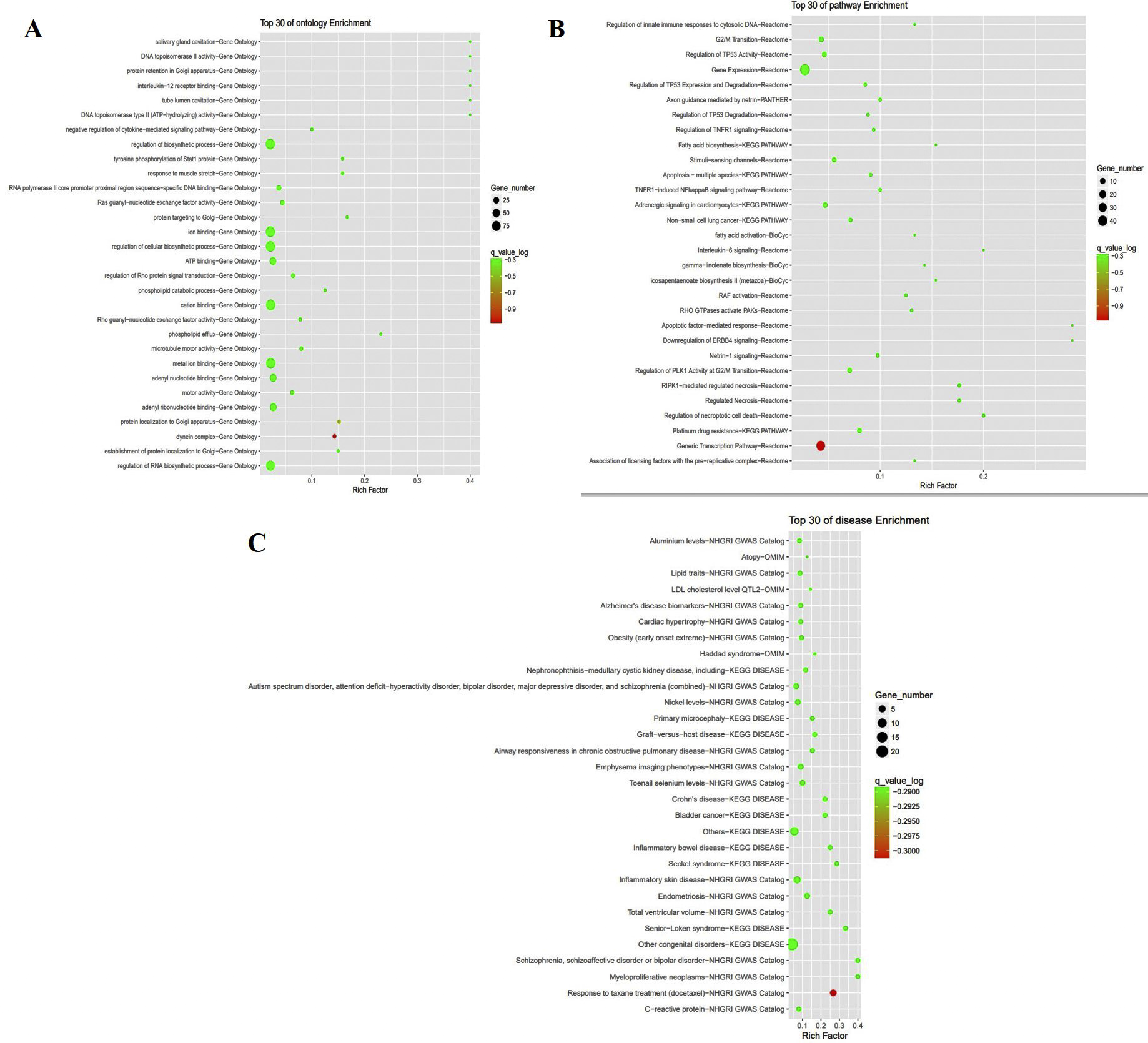

Supplement: Supplementary Figure 1 — Gene enrichment analysis in GEA. (A) Gene ontology enrichment analysis results. (B) Pathway enrichment analysis results. (C) Disease enrichment analysis results. [file Image1.tif]
